# Do Local Multidisciplinary Team Meetings in the UK Alter Management Recommendations for Invasive Treatment of Stress Urinary Incontinence?

**DOI:** 10.1007/s00192-025-06438-x

**Published:** 2025-11-15

**Authors:** Omar Kassab, Hui Ling Ong, Holly Bekarma, Inna Sokolova, Wael Agur, NHS Ayrshire, Arran Urogynaecology Multidisciplinary Team

**Affiliations:** 1 University of Edinburgh, 49 Little France Crescent, Chancellor’s Building, Edinburgh BioQuarter, Edinburgh, EH16 4SB UK; 2 The Newcastle Upon Tyne Hospitals NHS Foundation Trust, Tyne, UK; 3 NHS, Ayrshire & Arran, Ayrshire, UK; 4 University of Glasgow, Glasgow, UK

**Keywords:** Decision-making, Multidisciplinary team (MDT), Patient-centred care, Patient preferences, Stress urinary incontinence (SUI), Surgical management

## Abstract

**Introduction and Hypothesis:**

Traditional single-specialty approaches may be insufficient for managing surgical interventions in women with stress urinary incontinence (SUI). Limited data exist on the effectiveness of multidisciplinary teams (MDTs) in the decision-making process since NICE introduced the concept in 2013. This study evaluates the impact of a purpose-designed MDT on SUI management from 2017 to 2019.

**Methods:**

Monthly MDT meetings at a university hospital and a district general hospital reviewed cases of women considering invasive treatment for primary SUI. Attendees included gynaecologists, urologists, continence nurses, physiotherapists, and administrative staff. Discussions focussed on patient history, urodynamics, treatment preferences, and final recommendations. Data on age, parity, BMI, diagnoses, and treatment plans were analysed using SPSS Statistics (version 23).

**Results:**

A total of 123 women were discussed (mean age 54.5 years, mean BMI 30.8). Of 96 cases with MDT decisions, colposuspension was most recommended (46.9%), followed by bulking agent injection (37.5%) and autologous fascial sling (8.3%). MDTs altered management in 19/123 cases (15.4%), primarily shifting towards less invasive interventions. No mesh tape procedures were recommended despite availability. Among 86 women who identified priorities, cure from leakage (96.5%) and avoiding repeat surgery (47.7%) ranked highest.

**Conclusion:**

MDT discussions influenced treatment decisions by favouring conservative management options in many women. Patient preferences were fully considered during MDT discussions. Further studies should be conducted to assess long-term clinical outcomes following MDT recommendations.

## Introduction

Urinary incontinence is a highly prevalent and under-reported pathology worldwide. Recent summaries estimate that 423 million adults globally live with some form of urinary incontinence, with rates in women varying widely by age and definitions used [1]. Within female incontinence, stress urinary incontinence (SUI) is the most frequently reported sub-type [1]. Population estimates suggest that SUI affects roughly a quarter to nearly half of women over 30. This prevalence increases further with age, peaking in the 5th and 6th decades of life [2, 3].

SUI is caused by weakened pelvic floor muscles, either due to urethral hypermobility or urinary sphincter deficiency. This results in involuntary urinary leakage during activities that increase the intra-abdominal pressure such as sneezing or coughing. Women who engage in sports often complain of this type of incontinence [1, 3]. SUI is also attributed to age, obesity, oestrogen deficiency, cigarette smoking, as well as pregnancy and childbirth [4, 5]. Beyond leakage symptoms, SUI substantially affects quality of life, contributes to embarrassment, avoidance of social activities, reduced physical and occupational participation, and increased psychological distress. The condition also poses an economic burden related to continence products and healthcare utilisation [1–3]. These multidimensional consequences highlight the importance of optimising decision-making processes for women considering treatment.

According to current national and international guidelines, first-line management for SUI involves non-surgery with pelvic floor muscle training, duloxetine medical treatment, and continence pessaries [2, 3, 6]. If symptoms persist despite these treatments, then patients are referred to urogynaecology surgery.

Traditional single-specialty approaches, where a doctor and patient decide on a treatment option alone, might not be adequate anymore in choosing the surgical intervention most suited to a particular woman. Owing to shifting demographics—particularly an aging population and rising obesity rates—urinary incontinence is becoming increasingly complex, with a diverse range of associated comorbidities. Conditions such as pelvic organ prolapse and obstructive defecation commonly present alongside urinary incontinence in many patients [4, 7].

The complexities associated with surgical treatment pose additional challenges. Four procedures are available: colposuspension, autologous fascial sling, bulking agent injection, and mesh tape surgery. Currently, the mesh tape procedure is suspended in the UK and other countries over safety concerns, even though it continues to be recommend as a viable treatment option [6]. These surgeries are not without their complications, and up to a third of women may require re-operation for SUI. Moreover, the interval between successive operations tends to decrease over time, demonstrating the curative limitations of surgery [4].

In 2013, the National Institute for Health and Care Excellence (NICE) recommended that women considering SUI surgery should be reviewed by a dedicated multi-disciplinary team (MDT) composed of urogynaecologists, specialist nurses, physiotherapists, and potentially a colorectal surgeon [6]. The role of MDTs in urogynaecology for patient management and decision-making is, however, currently unclear.

Multi-disciplinary efforts have become standard in fields like oncology due to their numerous benefits. For instance, MDTs have been found to facilitate the standardisation of care, improve health outcomes, and reduce the incidence of suboptimal practices [7]. They can also contribute to more accurate tumour staging and neo-adjuvant/adjuvant treatment prescription [8]. These advantages suggest significant potential for improving the management of complex patient cases.

In urogynaecology, preliminary studies find that MDT meetings can lead to adjustments to patient management plans [9–11]. However, to our knowledge, no existing papers quantify how often treatment plans change after MDT meetings or describe the nature of those changes. This study’s primary objective is to determine the proportion of cases in which MDT involvement alters the initial management plan for women with SUI and to describe the direction and rationale of those choices. Secondary objectives are to compare MDT recommendations with patients’ stated values and treatment preferences.

## Materials and Methods

This was a retrospective observational cohort (service-evaluation) study of consecutive women with SUI considered for invasive treatment whose cases were discussed at monthly urogynaecology MDTs pre-COVID between 2017 and 2019.

A total of 36 meetings were held at a University Hospital (Crosshouse) and a local District General Hospital (Ayr), with around 40 surgical procedures for SUI performed every year. The MDT meetings were attended by gynaecologists, urologists, continence nurses, physiotherapists, urodynamicists, and administrative staff. No extra funding was used to host these meetings, which occurred at lunchtime at the good will of those involved. Sessions were scheduled for 60–120 min depending on the number of patients to be discussed.

During the meetings, each patient’s condition was meticulously reviewed, taking into account their presenting symptoms, medical history, individual concerns, treatment preferences, urodynamic findings, and the MDT’s proposed management plan. A local MDT proforma was adopted to standardise the discussions. This patient-centred approach facilitated discussion in situations where evidence did not indicate a single best treatment and several reasonable options existed.

In accordance with NICE guidelines, individuals were referred to MDT by gynaecologists, urologists, physiotherapists, and continence nurses once nonsurgical methods were exhausted [6].

Specifically, inclusion criteria for the study were patients with pure stress urinary incontinence or stress-predominant mixed urinary incontinence and being considered for invasive treatment after trial with conservative measures. Exclusion criteria were presentations to the MDT for conditions other than SUI, e.g. pelvic organ prolapse, surgical complications, bladder pain syndrome, urgency-predominant incontinence, Fowler’s syndrome, and other complex urogynaecological conditions.

The majority of patients considering surgery were *primary* SUI, except for six women who presented for discussions of their *recurrent* SUI, mostly following mesh tape procedures.

As part of the routine pre-MDT pathway, patients were asked to note down their preferred surgical option (initial treatment preference) and the top three values influencing their decision using the SUI-PDA [12]. The primary analysis included all women discussed by MDT (*n* = 123). To align MDT recommendations with patient values, we only included respondents who completed the “top three values” field (*n* = 86). For the initial treatment preference comparison, we analysed respondents who completed that item (*n* = 102). For summaries of final MDT recommendations, we included respondents with a recorded MDT decision (*n* = 96).

Patient data was then collected and anonymised. To examine the role of MDT in SUI management, demographics (age, parity, BMI), clinical details (primary diagnosis, urodynamics, symptoms questionnaires), patient preferences, and final recommendations from MDT were assessed using SPSS Statistics (version 23). Since this was a retrospective cohort analysis and evaluation of the standard MDT service delivered to our patients, no ethical approval was needed. No procedures beyond usual care were introduced and no identifiable data left the clinical teams. For this analysis, a de-identified dataset was created prior to export and direct identifiers were removed. Individual consent for analysis of anonymised routine data was not required.

The analysis in this study was descriptive, with the main endpoint being the proportion of cases in which the MDT recommendation differed from the initial plan, and additional endpoints including direction/rationale of changes, alignment with patient-stated values/preferences, and waiting-time intervals.

## Results

The local MDT discussed a median of 5 patient conditions (range 4–10) each monthly session, assessing a total of 123 SUI cases over the 3-year period. Three to four SUI patients were discussed at each meeting. The remaining patient presentations were due to pelvic organ prolapse, pure overactive bladder symptoms, urgency-predominant mixed urinary incontinence, and other complex urogynaecology conditions such as Fowler’s syndrome. These were excluded from our analysis.

Of the 123 women discussed, initial preference was available for 102, a final MDT decision was recorded for 96, and top three values were reported for 86. Only three patients required a second discussion after previously unavailable information (e.g. video urodynamics) became available.

The average age was 54.5 (range 35–85 years), the median parity was 2 (range 0–7) and the mean BMI was 30.8 kg/m^2^ (range 21–49). No underweight women presented with symptoms during the study period. The highest incidence of incontinence appeared to be within the age range of 49–53 years, reflecting the time of menopause, however, we did not record the duration of SUI symptoms prior to being assessed by MDT. The parity also demonstrated that childbirth was common in our population. Only two patients were null gravida.

Most women (90.2%) presented with stress-predominant mixed urinary incontinence. Twelve women (9.8%) presented with pure-stress urinary incontinence. No urgency-predominant cases were included.

On average, 3 gynaecologists, two urologists, 5 continence nurses, 5 physiotherapists, and 1 administrator attended the multidisciplinary meetings. The median duration between patient referral to the MDT and the scheduled meeting date was 65 days. Most women were referred by gynaecologists/urogynaecologists (*n* = 90), followed by urologists (*n* = 33).

Table 1 showed the frequency of patient choices regarding the 4 main SUI procedures for 102 out of 123 respondents. The most favoured procedure amongst women was colposuspension (60.8%) followed by bulking agent injection (28.4%) and autologous fascial sling (10.8%). None of the women respondents showed preference for conservative (nonsurgical) treatment.

**Table 1:** Comparing the frequency and percentage of patient-selected treatments—colposuspension, bulking agent injection, autologous fascial sling, mesh tape surgery, or no surgery—with those recommended by the MDT

	Patient choice frequency	Patient choice percent	MDT choice frequency	MDT choice percent
Colposuspension	62	60.8	45	46.9
Bulking agent injection	29	28.4	36	37.5
Autologous Fascial Sling	11	10.8	8	8.3
No surgery	0	0	7	7.3
Mesh tape surgery	0	0	0	0

**Fig. 1:**
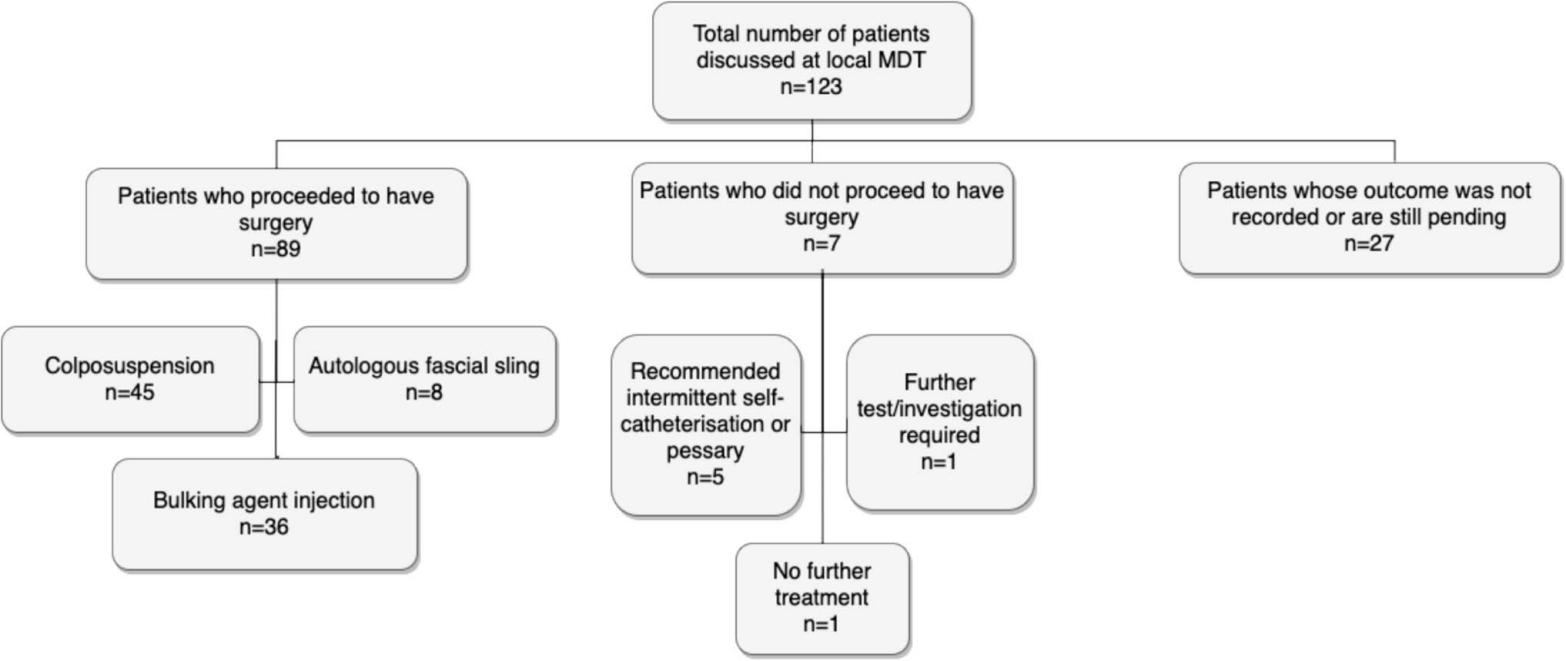
The management outcomes of patients discussed at local MDT (*n* is the number of patients)

MDT decisions were available for 96 of the 123 patients (78%) and showed a similar pattern, with colposuspension being the most frequently recommended procedure (46.9%), followed by bulking agent injection (37.5%) and autologous fascial sling (8.3%). However, in contrast to the women—who all preferred surgical management—the MDT recommended no surgical intervention for seven patients (7.3%). Instead, these individuals were advised to undergo further investigations (e.g. video urodynamics) or pursue conservative management (see Fig. 1).

Despite the availability of mesh tape procedures in a nearby hospital, the MDT did not suggest such surgery in any patient. The recommendations for surgery were based on symptom severity, patient comorbidity, as well as consideration of patient choice.

On average, it took 88 days after MDT before women underwent their respective surgeries. While the average period of 65 days from referral to MDT discussion could be reduced by more frequent meetings, the average period of 88 days to undergo their procedure was largely dependent on our local waiting times list.

In 19 cases (15.4%), the MDT recommended a course of action different from the women’s initial preferences. Of these, the MDT advised bulking agent injection in 12 cases (63.2%) and nonsurgical management (either conservative treatment or further investigation) in six cases (31.6%). In only one instance (5.3%) did the MDT recommend switching from one major surgical procedure to another, changing the plan from colposuspension to autologous fascial sling due to low urethral pressure. Comparative analysis would not have been meaningful due to the relatively low number of instances where MDT recommendations were at variance with patient preferences.

Moreover, all patients documented the key values that influenced their choice of SUI procedure. Among the 15 possible values, 86 women identified their top three. “Cure from leakage” was the most frequently selected, with 83 out of 86 women (96.5%) including it in their top three priorities. The second most important value was “avoiding repeat surgery in the future,” chosen by 41 women (47.7%), and the third most important was “avoiding mesh complications,” selected by 30 women (34.9%). See Table 2 for details.

**Table 2:** Top three factors influencing patients’ choice of SUI procedure

	Frequency	Percentage (%)
Cure from leakage	83	96.5
Avoid repeat surgery in the future	41	47.7
Avoiding mesh complication	30	34.9

None of the patients opted for mesh tape surgery, primarily due to safety concerns. Most women accepted the MDT recommendations, even if they were contrary to their initial preference. Among the 19 women whose management plans were altered by the MDT, only one patient disagreed with the MDT decision. She declined the advised bulking agent injection and instead chose to proceed with an autologous fascial sling, citing concerns about the potential need for repeat procedures and the limited likelihood of complete leakage resolution with bulking agent.

## Discussion

### Principal Statement

Our MDT refined the management of 19/123 SUI cases (15.4%). Reported change rates across pelvic floor/urogynaecology MDT cohorts vary widely, largely due to case-mix and how “management change” is defined. Studies with broader indications (e.g. pelvic organ prolapse, surgical complications, and bladder pain syndrome) report higher figures—Ong 41.7%, Wales 31%, Ghopinath & Jha 29.3%, Pandeva 20%—so direct comparison with our SUI-only cohort is limited [9–11, 13]. The relatively lower rate of 15.4% in this study is likely due to our narrower selection of exclusively SUI presentations.

In this study, the local MDT favoured less invasive approaches—such as bulking agent injection or nonsurgical management—over more invasive procedures (e.g. colposuspension and autologous fascial sling) in 18 of the 19 cases where the initial management plan was altered. According to the latest national report from the British Society of Urogynaecology (BSUG), interventions with the lowest postoperative complication rates, as measured by 30-day readmission, were bulking agent injections (0.1%), followed by colposuspension (1.75%), and the autologous fascial sling (4%) [14]. These data indicate that the MDT’s recommendations aligned with a risk-conscious, evidence-based approach, prioritising patient safety by favouring procedures associated with lower complication rates when clinically appropriate.

This trend of preferencing less invasive treatments has been reported before; for instance, by Gopinath and Jha, who saw their MDT offer laparoscopic colposuspension over autologous fascial sling for a 66-year-old woman [13].

Our MDT suggested an escalation in intervention only once by choosing the autologous fascial sling instead of colposuspension. While the fascial sling is associated with higher morbidity, it also offers greater efficacy [15], particularly in cases where urethral pressures are low. This exception underscores the MDT’s capacity for sophistication by utilizing patient-specific factors such as urethral pressures to reach the most suitable management option.

Women in our study were generally older, had a higher body mass index (BMI), and greater parity, which are all risk factors for SUI development [4]. It is well-established that developed countries are experiencing an ageing population, largely due to advances in medicine and healthcare [16]. Thus, the demand for surgical intervention among women is likely to increase, particularly given that the lifetime risk of undergoing surgery for incontinence or prolapse surgery is 11.1% by age 80 [4]. In this context, MDTs play a crucial role in safeguarding against unnecessary surgical procedures, thereby reducing the risk of postoperative complications and long-term morbidity.

Among the surgical options evaluated in our study, colposuspension was the most frequently selected procedure, followed by bulking agent injection and, lastly, autologous fascial sling, as preferred by both patients and the MDT. This contrasts with the 2021 trends from the BSUG report, where bulking agent injection accounted for 68% of all reported surgeries, colposuspension for 20%, and autologous fascial sling for 11.5% [14].

Given that most women in this study prioritized a definitive solution for urinary leakage and nearly half expressed a desire to avoid repeat surgeries, the preference for colposuspension over bulking agents is clear. This choice aligns with the patients’ goals of achieving long-term SUI resolution and minimizes the need for future interventions. A similar sentiment was reported by Brazzelli et al. who, after studying the questionnaire responses of 789 women, found that patients preferred “surgical treatments associated with no pain or mild chronic pain and shorter length of hospital stay as well as those treatments that have a smaller risk for urinary symptoms to reoccur after surgery” [17].

Consequently, the discrepancy between the trends observed in our MDT’s decisions and those reported in BSUG national data may be attributed to the incorporation of patient preferences and values, which were available to the MDT during its deliberations and played a central role in guiding recommendations. This patient-centred approach is likely to enhance satisfaction and engagement, as reflected by the high level of acceptance of MDT recommendations among the women in this study.

Meanwhile, the autologous fascial sling, like the BSUG, was the least frequently chosen procedure in our study. This may be due to its higher rate of complications and morbidity, as well as its functional similarity to the mesh tape procedure, as both techniques utilize slings to provide urethral support [14]. Given the strong emphasis among patients on avoiding mesh-related complications, the limited adoption of the autologous fascial sling is understandable. In 2018, the UK government imposed restrictions on the use of mesh tape surgery due to safety concerns, limiting its application to exceptional cases where no viable alternatives exist [18]. Since most patients in our cohort were surgery-naive, the multidisciplinary team found no clinical justification to recommend mesh tape procedures. Furthermore, none of the patients expressed interest in this option because of worries about potential side-effects.

### Strengths of Our Study

As far as we are aware, none of the earlier studies who reported adjustments to patient management following urogynaecological MDTs have described in detail the type of changes being made, relative to patient values and preferences. This study was the first to highlight how MDTs determined the best intervention for each patient, opting for less invasive procedures in instances when the risks of surgery did not outweigh the benefits.

Furthermore, the composition of our MDT team met and exceeded the NICE guideline requirements of having two consultants, one specialist nurse, and one specialist physiotherapist [6]. We included gynaecologist consultants in our study because of their expertise in pregnancy-related SUIs. We also included urologists who specialized in complex pelvic floor disorders and surgical treatment. Finally, we involved continence nurses and physiotherapists because of their contributions to nonsurgical decisions like pelvic floor muscle training and lifestyle modifications. Their involvement also facilitated coordination among team members and ensured continuous patient monitoring and follow-up. Lastly, although not mandated by NICE guidelines, the inclusion of an administrator was found to enhance team efficiency by managing scheduling and ensuring effective time management during meetings.

Since this was a urogynaecology MDT rather than a pelvic floor one, a colorectal surgeon was not required. However, they were invited if women with significant bowel problems were discussed.

### Limitations of Our Study

It remains unclear whether the MDT recommendations led to improved outcomes or not. Future studies should compare prognostic metrics (e.g. patient satisfaction, long-term cure rates, treatment side-effects) in patients who had their cases discussed at MDT versus patients who did not. However, it is reasonable to assume based on our results that MDT decisions will be safer and more reliable since they are the result of collective conversation instead of the opinion of a single specialist. The lack of malpractice suits involving MDT settings further corroborates this notion [13].

In addition, the lack of administrative support in our study led to variable consistency in documenting the MDT decisions, which contributed to our attrition rate of 22%. While such a rate may have affected our results, a similar preference pattern, with colposuspension being the most frequently recommended procedure, followed by bulking agent injection and autologous fascial sling, is consistent with our overall clinical experience.

Finally, our analyses were descriptive by design, aiming to map MDT decision-making rather than infer causality. Given the modest sample size, adjusted comparisons would risk over-interpretation. More rigorous statistics are needed in later studies (preferably prospective and multicentre) to test associations.

### Clinical Applications

SUI is a prevalent condition, particularly amongst older postmenopausal women with declining oestrogen levels and urogenital tissue compromise [19]. The UK has an ageing population; in 2022, 19% of the population was 65 or over. This is projected to increase to 27% by 2072 [16]. As the prevalence of SUI is expected to rise alongside an ageing country, there is a need to optimise our approach towards patient management. Many available procedures for SUI have deleterious side-effects, with reoperation rates reaching up to 21.3% within 10 years [20]. Other surgeries, like the mesh tape, have been the subject of litigation due to concerns over their invasiveness [18]. MDTs provide a structured framework for enhanced decision-making, which may facilitate the selection of less invasive surgeries, minimize unnecessary procedures, and reduce the risk of long-term complications.

One barrier to adopting MDTs for urogynaecology has been the cost, since MDTs in general require lots of time and specialists. Models from oncology have previously provided estimates for the cost of MDT implementation (e.g. £87.41 per patient) [13]. However, comparable data for non-oncology MDTs, including urogynaecology, remain limited and no consensus exists regarding their cost-effectiveness [11]. Establishing routine SUI-specific MDTs would likely require additional funding or resource allocation. In our setting, the MDT discussions were undertaken within existing job plans and incurred no additional cost to the hospital.

In the long-term, the lack of financial incentive may discourage many practitioners and hospital administrators from hosting such meetings. It has already been reported by Balachandran et al. that 70% of members of the British Society of Urogynaecology oppose the NICE recommendation for MDT prior to SUI surgery due to the lack of an evidence-base [21]. Fiscal challenges can further solidify this opposition.

In our study, MDTs selected less invasive treatments like self-catheterization or pessary, which significantly cost less than SUI surgeries. Therefore, the financial burden of MDT implementation may be offset by cost savings associated with these lower-risk alternatives, which generally have fewer complications.

While this is promising, the decision not to operate should be balanced against the long-term costs associated with temporary and non-curative treatments (e.g. bulking agent injection).

Aside from finances, MDTs may further prolong the waiting times, since each patient must be evaluated by an MDT service before being eligible for treatment. In the current study, MDTs were held once every month and resulted in waiting times of approximately 2 months. However, this might be improved if more frequent meetings were held, for instance every week, significantly reducing the waiting times to 2–14 days [13].

Unfortunately, resource-strained hospitals might find the idea of weekly meetings too difficult, since personnel and funding are limited. Other options would be to reserve MDTs for complex or atypical cases of urinary incontinence, while routine cases could follow a predesigned hospital pathway set by an initial MDT. This model was implemented by the Southern Region Pelvic Floor MDT, who found it beneficial [22]. However, inclusion criteria for such MDTs will need to be determined.

In short, adequate NHS funding is essential to support implementation of MDTs nation-wide. Demonstrating the efficacy of MDTs through research may help obtain this funding and encourage NHS managers to integrate these services into routine practice. In the past, the NHS demonstrated adaptability by restructuring its oncology services to incorporate MDTs for head, neck, and lung cancers. This led to improved patient survival and adherence to clinical standards [23]. Thus, it is not inconceivable for the NHS to repeat the same experience with SUI patients.

Although our findings reflect practice in two UK hospitals, the concept of multidisciplinary review is generalisable to a diverse array of healthcare systems, who might not face the same economic limitations as the NHS. Therefore, it might be more feasible for other countries to host multidisciplinary team meetings.

Unfortunately, the international community does not seem to have reached a consensus yet on how to define MDT care, which specialists to involve, or how to conduct the meetings [23]. This lack of agreement could hamper further research, application, and evaluation of MDTs. The NICE recommendations on MDT composition provide a reasonable framework with potential for application outside the UK.

### Recommendations

Studies on the long-term impacts of MDT choices compared to traditional consultations should be undertaken. Further research is needed to determine the best method of conducting MDTs in terms of meeting duration, frequency, and structure. It would be valuable as well to explore whether disagreement between patient values and MDT recommendations influenced patient satisfaction. Finally, there is little evidence on which proformas to use for standardised meeting discussions. All these gaps highlight important avenues for future research.

## Conclusion

Local multidisciplinary team discussions have changed the proposed management for women considering surgery for stress urinary incontinence. Most changes were to a less-invasive intervention. Patient values and preferences have an important role to play in MDT management. Future work should prospectively evaluate whether MDTs improve patient-centred outcomes (continence, complications, satisfaction), decision quality, and time-to-treatment. This should ideally be done in multicentre and international cohorts.

## Data Availability

Data are available upon request.

## Declarations

### Conference Presentations

International Continence Society 2020 Las Vegas/Online: https://www.ics.org/2020/abstract/10.
